# ABPS: An R Package for Calculating the Abnormal Blood Profile Score

**DOI:** 10.3389/fphys.2018.01638

**Published:** 2018-11-21

**Authors:** Frédéric Schütz, Alix Zollinger

**Affiliations:** ^1^Bioinformatics Core Facility, SIB Swiss Institute of Bioinformatics, Lausanne, Switzerland; ^2^Center for Integrative Genomics, University of Lausanne, Lausanne, Switzerland

**Keywords:** code:R, anti-doping, ABPS, blood doping, machine learning

## Abstract

The Abnormal Blood Profile Score (ABPS) is used to identify blood doping in sport. It combines seven hematological markers, including hemoglobin level, reticulocytes percent, and haematocrit level, using two different machine learning algorithms in order to create a single score that has a better ability to identify doping than each parameter taken alone. The resulting score allows the detection of several types of doping using a single score and is part of the current Athlete Biological Passport program managed by World Anti-Doping Agency (WADA). We describe ≪ ABPS ≫, an R package that allows the calculation of this score. This is the first software implementation calculating this score that is released publicly. The package also contains functions to calculate the OFF-score (another score used for detection of doping), as well as several test datasets. The package is useful for laboratories conducting anti-doping analyses and for researchers working on anti-doping research projects. In particular, it has been successfully used in projects estimating the prevalence of blood doping.

## Introduction

The Abnormal Blood Profile Score (ABPS) is one of several tools used to identify blood doping in elite athletes. Originally developed at the Swiss Laboratory for Doping Analyses (LAD) in Lausanne (Sottas et al., 2006), it combines 7 hematological markers (reticulocytes percent, hemoglobin level, haematocrit level, red blood cell count, mean corpuscular volume, mean corpuscular hemoglobin, mean corpuscular hemoglobin concentration) into a single score. According to the original ABPS publication, this combined score is more sensitive (for the same level of specificity) to doping than any of these blood parameters taken separately; in particular, the ABPS alone allows the detection of several types of blood doping (Sottas et al., 2006). ABPS is based on two different classification techniques, a naive Bayesian classifier and an SVM (Support Vector Machine). The two models were trained using a database of 591 blood profiles (including 402 control samples from clean athletes and 189 samples of athletes who abused of an illegal substance); the two scores were then combined using a method called ≪ ensemble averaging ≫ to obtain the final score. The resulting ABPS values are unitless; based on a large collection of test results (>3,000 samples), they typically range between −2.35 and 1 (95% confidence interval, with a mean of −0.67 and a standard deviation of 0.87); more detailed reference distributions and the effect of different factors (sex and age of athlete, type of sport, etc) on the score is described elsewhere (Robinson et al., 2018). While ABPS is not a primary marker of doping, it has been used as corroborative evidence (CAS, 2012) and it is part of the Athlete Biological Passport (ABP) program managed by the World Anti-Doping Agency (WADA; Sottas et al., 2011a). Assuming that the testing was conducted according to the WADA guidelines (WADA, 2018), scores between 0 and 1 indicate a possible suspicion of doping (CAS, 2012); a score above 1 should be found only in 1 in 1,000 clean male athletes.

Several versions of ABPS scoring have been developed over the years, using up to 12 different blood parameters (Sottas et al., 2010); however, the most commonly used version is based on the 7 parameters described above, which can be obtained “on-line” on a portable analyzer. The 7 parameters were selected following a comparison of the performances of models with different numbers of parameters. The original ABPS implementation was created using the Matlab programming language, and WADA maintains a Java version that is used within the ADAMS (Anti-Doping Administration and Management System) database and the Athlete Biological Passport program. However, these versions are only available to anti-doping organizations recognized by WADA.

## The R package ABPS

The ABPS package allows users to compute the Abnormal Blood Profile Score using the R statistical software (R Core Team, 2017). It is the first such software implementation that is released publicly. The R software was implemented using the original Matlab software as a reference; in particular, no new model fitting was performed, as the original parameters calculated in Matlab for the naive Bayesian and the SVM classifiers were reused in the R code. As a result, the package does not require the original database of blood profiles, which is not publicly available. The concordance of results obtained using the Matlab software and the R implementation was checked using a series of test data, some of which are included in the package (see section ≪ datasets ≫ below for more details).

**Figure 1:**
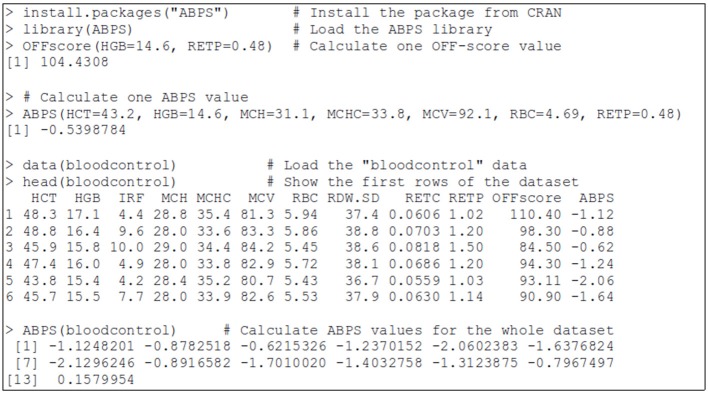
A sample session using the ABPS package within R. During this session, the package is installed, loaded, the ≪ bloodcontrol ≫ dataset is loaded, and some calculations of ABPS and OFF-score are performed.

The ABPS function available in the package requires the user to provide values for the seven hematological markers for one or several samples, and will then calculate and return the corresponding score or scores. The markers can be specified either as a single data frame (the basic structure for managing data in R) containing the seven parameters, or by specifying separately the seven following variables (the expected units are indicated): HCT [haematocrit level, in [%]], HGB [the hemoglobin level, in [g/dL]], MCH [the mean corpuscular hemoglobin, in [pg]], MCHC [the mean corpuscular hemoglobin concentration, in [g/dL]], MCV [the Mean corpuscular volume, in [fL]], RBC [the red blood cell count, in [10^6^/ μL]], RETP [the reticulocytes percent, in [%]]. A short example of use is shown on Figure 1; detailed information and examples of use are provided in the help page of the function.

The package also provides a function for calculating the OFF-score (Gore et al., 2013), another indirect indicator of blood doping. Also called “OFF-hr score” or “stimulation index” (Zorzoli, 2011), the OFF-score is part of the Athlete Biological Passport and is routinely used to identify athletes who use a substance prohibited by anti-doping rules (CAS, 2016). It combines the hemoglobin level [HGB, [g/dL]] with the reticulocytes percent (RETP) using the formula

$$\begin{array}{l} \text{OFF}-\text{score}=\text{HGB}\times 10-60\times \sqrt{\text{RETP}} \end{array}$$

The original publication (Gore et al., 2013) provides thresholds for different populations; OFF-score values typically range between 85 and 95 and in the worst case scenario (a male athlete living at low altitude), values over 133 are considered to be evidence of doping (Marrocco et al., 2012). Note that the original publication assumed that the hemoglobin level is specified in g/L; as hemoglobin levels are generally specified in g/dL (for example, in the ADAMS database) and to ensure coherency with other functions within the package, our OFF-score function also expects hemoglobin levels to be specified in g/dL, as for the ABPS calculation, and it will then convert the units internally (as shown by the presence of a multiplication by 10 in the formula above). The OFF-score function will emit a warning if the units seem wrong.

## Datasets

In addition, the ABPS package provides several datasets linked to blood doping. The first dataset, named ≪ bloodcontrol ≫, contains blood parameter data measured on 13 individuals, provided by the Swiss Laboratory for Doping Analyses (LAD) in Lausanne. These samples are assumed to represent normal population, and were not used for fitting the original ABPS model.

The second dataset, named ≪ blooddoping ≫ contains a series of 13 measurements of blood parameters conducted over several years on a female athlete who was later convicted of repeated doping on the basis of this data (CAS, 2012). The published data contains some errors (in particular, some values were swapped between samples) which were discovered while creating the package and confirmed by WADA. The package provides a corrected version of the dataset, as well as a detailed list of the changes that were made.

For both datasets, the OFF-scores and ABPS values provided were computed by WADA software. The R package can thus be tested by comparing the results it produces with the ones computed by WADA.

## Documentation and tests

The package comes with extensive documentation, in particular help pages, detailing both the available functions and datasets, as well as a set of unit tests which verify that the code works as it should and that it yields the expected results on the provided datasets. The source code is commented in details and the package passes all the checks provided by R without any error, warning or note.

## Package usage

The functions provided by the package can be used by laboratories conducting anti-doping analyses who may want to calculate ABPS values based on measurements of blood parameters they have performed, or by people interested in anti-doping who want to examine and understand how the ABPS scheme works, and how sensitive it is to changes in one blood parameter or another.

However, the most likely use of the package (and the primary motivation for its creation) is in anti-doping research. As an example, the ABPS was used in the past to estimate the prevalence of doping in different populations of athletes (Sottas et al., 2011b). In this context, ABPS allows the researcher to work with a single, combined, parameter instead of seven different markers. The comparison of its distribution for different groups of interest can be used to estimate the prevalence of doping. Further research on this topic has been conducted using this R package and will be described elsewhere.

## Limitations of the package

As described above, the package allows a user to calculate the same ABPS values as available, for example, in the ADAMS database, thus providing a way to faithfully reproduce these results. However, as a consequence, the package inherits any potential shortcomings that the original ABPS algorithm may have. In particular, the original training dataset was based on athletes who received rhEPO injections (Sottas et al., 2006), and it may not be representative of other doping methods. For example, the ABPS will lose sensitivity with either volume expansion (IV or hyper hydration) or sub-micro dose EPO masking. As the authors of ABPS note (Sottas et al., 2006), the algorithm may need to be updated using new datasets when new blood doping methods are discovered.

The ABPS values are dependent on the blood analyzer used to generate the measures and are sensitive to bad pre-analytical and analytical conditions which have an impact on one or more of the seven hematological markers, such as variation in instrument calibration; the protocols for collection, transport and analysis given in the WADA Athlete Biological Passport Operating Guidelines (WADA, 2018) should be used to guarantee a good interpretation of ABPS values. In addition, the distribution of the ABPS values, like the distribution of each of the seven hematological markers, is dependent on the population studied; different groups of athletes (for example, males vs. females) may display different distributions of ABPS values than the one obtained for the reference population on which the ABPS is based. For some usages of ABPS (for example, in a study of the prevalence of doping), it is thus advised to apply correction factors for the different populations. A future version of the package will include examples of such corrections, as described elsewhere (Robinson et al., 2018). It is worth noting that the ABPS algorithm restricts the possible input values to a range which depends on the values observed in the original reference dataset. If a value is outside this range, it will be modified to the minimum of maximum accepted value; for example, any HGB value lower than 12.9 or 18.2 will be modified to 12.9, respectively 18.2. The exact range for each parameter is indicated in the package; a warning will be printed if an input value is outside the allowed range.

## Availability of the package

The R package is available either from CRAN (Comprehensive R Archive Network, https://cran.r-project.org/package=ABPS) or from a git server (https://gitlab.isb-sib.ch/BCF/ABPS). It is distributed under a free license, the GNU General Public License (version 2 or later).

In term of dependencies, the ABPS package requires only one package external to the base R system: kernlab, which is also available freely (licensed under the GNU GPL version 2) on CRAN.

## Author contributions

FS wrote the R package and the paper. AZ wrote part of the R package and approved the paper.

### Conflict of interest statement

The authors declare that the research was conducted in the absence of any commercial or financial relationships that could be construed as a potential conflict of interest.

## Abbreviations

ABPS: Abnormal Blood Profile Score
ADAMS: Anti-Doping Administration and Management System
LAD: Swiss Laboratory for Doping Analyses
WADA: World Anti-Doping Agency.
